# Hierarchical spatial sampling reveals factors influencing arbuscular mycorrhizal fungus diversity in Côte d’Ivoire cocoa plantations

**DOI:** 10.1007/s00572-020-01019-w

**Published:** 2021-02-27

**Authors:** Cristian Rincón, Germain Droh, Lucas Villard, Frédéric G. Masclaux, Assanvo N’guetta, Adolphe Zeze, Ian R. Sanders

**Affiliations:** 1 Department of Ecology and Evolution, University of Lausanne, Lausanne, Switzerland; 2 Laboratoire de Génétique, UFR Biosciences, Université Félix Houphouët-Boigny, Abidjan, Côte d’Ivoire; 3 Laboratoire de Biotechnologies Végétale Et Microbienne, Unité Mixte de Recherche Et D’Innovation en Sciences Agronomiques Et Génie Rual, Institut National Polytechnique Félix Houphouet-Boigny, Yamoussoukro, Côte d’Ivoire

**Keywords:** Arbuscular mycorrhizal fungi, Cocoa, Diversity, Community composition

## Abstract

While many molecular studies have documented arbuscular mycorrhizal fungi (AMF) communities in temperate ecosystems, very few studies exist in which molecular techniques have been used to study tropical AMF communities. Understanding the composition of AMF communities in tropical areas gains special relevance as crop productivity in typically low fertility tropical soils can be improved with the use of AMF. We used a hierarchical sampling approach in which we sampled soil from cocoa (*Theobroma cacao* L.) plantations nested in localities, and in which localities were nested within each of three regions of Côte d’Ivoire. This sampling strategy, combined with 18S rRNA gene sequencing and a dedicated de novo OTU-picking model, allowed us to study AMF community composition and how it is influenced at different geographical scales and across environmental gradients. Several factors, including pH, influenced overall AMF alpha diversity and differential abundance of specific taxa and families of the Glomeromycotina. Assemblages and diversity metrics at the local scale did not reliably predict those at regional scales. The amount of variation explained by soil, climate, and geography variables left a large proportion of the variance to be explained by other processes, likely happening at smaller scales than the ones considered in this study. Gaining a better understanding of processes involved in shaping tropical AMF community composition and AMF establishment are much needed and could allow for the development of sustainable, productive tropical agroecosystems.

## Introduction

Arbuscular mycorrhizal fungi (AMF) are obligate biotrophs that form symbioses with the roots of the majority of plant species. This symbiosis is of fundamental ecological and agricultural importance because the fungi improve plant nutrient acquisition, especially phosphate. Likewise, AMF strongly influence soil structure and ecosystem functioning (Rillig and Mummey 2006; Powell and Rillig 2018), crop productivity (Ceballos et al. 2019; Zhang et al. 2019), and the diversity of plant communities (van der Heijden et al. 1998, 2015).

Recent biogeographic studies on AMF have shown that most AMF taxa present global distribution patterns (Davison et al. 2015) and that even identical genotypes of some AMF taxa occur in distant geographical locations, sometimes across continents (Savary et al. 2018). Patterns of AMF abundance, diversity, and composition have been found to be highly heterogeneous across multiple spatial and temporal scales (Bahram et al. 2015). For example, AMF communities were found to be phylogenetically clustered at scales of few meters (Horn et al. 2014) but driven by the local environment at a landscape scale (Hazard et al. 2013). For many soil-borne organisms, such patterns have been attributed to soil nutrient distribution (Oehl et al. 2005; Liu et al. 2014) and other edaphic conditions (Jansa et al. 2014). However, because AMF are obligate symbionts, it has been suggested that AMF assemblages co-vary with those of plant communities (Zobel and Öpik 2014; López-García et al. 2017).

In ecosystems, plant and AMF communities tend to be structured in a hierarchical way in which interactions between plants and AMF can occur at multiple scales (e.g., plant community, AMF population, AMF community within the plant). A major challenge in ecology is to understand the extent to which processes at one scale (e.g., within a population) affect patterns and processes at another (e.g., across the community). Explicit consideration of spatial scales in AMF community studies is rare (Vályi et al. 2016).

Despite their global distribution and widely recognized importance, the majority of the data available concerning the structuring of AMF communities and taxa distribution come from temperate areas (Öpik et al. 2010). Recent efforts have been made to bridge this gap. However, most of these studies have focused on extraction of AMF spores from the soil and subsequent morphological identification (Abdelhalim et al. 2014; de Mello et al. 2018; Sousa et al. 2018; Marinho et al. 2019; Solís-Rodríguez et al. 2020) rather than using DNA sequences for identification. Large degrees of variation in spore morphology have been reported even within an AMF species (Walker and Vestberg 1998). Also, many AMF may grow only vegetatively for long periods of time without producing spores (Helgason et al. 2002). Molecular analyses of AMF communities provide a way around these obstacles as they have the potential to elucidate AMF taxa in field samples independently of morphological criteria (Öpik et al. 2010). Some studies conducted in tropical regions using molecular techniques have reported the presence and abundance of AMF taxa. However, they have not addressed factors influencing community structuring processes (Senés-Guerrero and Schüßler 2015; Séry et al. 2018; Peña-Venegas and Vasco-Palacios 2019; Sarr et al. 2019). The lack of knowledge of tropical AMF community distribution and biogeography hinders our ability to harness the ecological services of AMF towards sustainable agriculture.

It recently has been shown that fungal phyla follow the typical latitudinal diversity gradient in which diversity increases from the poles to the tropics. However, this trend did not appear for fungal functional diversity, which was lowest in temperate biomes (Bahram et al. 2018). This highlights the possibility that tropical areas host a great taxonomic and functional diversity of AMF which remains understudied. Furthermore, there are no published hierarchical studies to understand factors shaping tropical AMF communities and whether they follow patterns observed in studies at different spatial scales in better-studied temperate ecosystems. The study of processes determining the structuring of AMF communities in tropical areas gains special relevance as most tropical soils have inherently low nutrient availability for plants, with high phosphate fixation rates due to low pH and high aluminum concentrations, which often leads to low crop productivity. The use of AMF has been shown to be an economically viable alternative to increase food production of some crops in tropical systems (Sieverding 1990; Ceballos et al. 2013, 2019). The application of AMF in cocoa (*Theobroma cacao* L.) is of great interest because AMF have been shown to confer several advantages such as growth increases (Snoeck et al. 2010), protection against pathogens (Tchameni et al. 2012), and reduced heavy metal uptake (Gramlich et al. 2017). Côte d’Ivoire is the world’s largest producer of cocoa, supplying 38% of cocoa used for chocolate manufacture. Historically, in Côte d’Ivoire, when productivity of a plantation decreases due to soil nutrient depletion, plantations are abandoned, inducing severe economic effects (Tondoh et al. 2015).

In this study, we used a hierarchical sampling approach in which we sampled soil from two cocoa plantations nested in localities, and in which several localities were nested within each of three cocoa production regions in Côte d’Ivoire. We chose plantations that had the area in a radius of several meters around the trees kept free of weeds with a ground cover of cocoa leaves and no other plants. Thus, sampling the soil around cocoa trees mostly avoided the environmental filtering rendered by diverse plant communities over AMF assemblages. This allowed us to best study the influence of geographical and environmental variables on AMF community composition. Our aim was to study AMF community composition and how this is influenced at different geographical scales and across climatic and environmental gradients. Besides investigating the factors influencing AMF community composition at different scales, this study also intends to contribute to bridging the gap concerning knowledge of tropical AMF communities associated with plantation environments.

## Materials and methods

### Sampling sites and strategy

We used a hierarchical strategy to sample soil in three regions in Côte d’Ivoire with several localities within each region and two plantations within each locality (Fig. S1). These three regions represent the most important cocoa production areas in Côte d’Ivoire. The first, the Nawa region, the highest cocoa production area, is characterized by an average annual rainfall of 2200 mm, with two rainy seasons (March–June and September–November) (Goula Bi Tie et al. 2007). In this region, soil samples were taken from two cocoa plantations in each of three localities: namely, Petit-Bondoukou (PBK), Koda-Centre (KDA), and Kipiri Grand-Zatry (KPI). The trees sampled in each of the plantations were of similar ages but the exact age since planting for each plantation was not available. The second region, the San Pedro region climate, is characterized by an annual rainfall between 1600 and 2400 mm, with two rainy seasons (April–July and October–November) (Goula Bi Tie et al. 2007). In this region, soil samples were taken in two cocoa plantations in each of three localities, namely, Camp Conakry (CKY), Gabiadji (GBI), and Touih (TH). The third region, the Gôh region climate, is marked by an annual rainfall varying from 1200 to 1600 mm, and by the alternation of two rainy seasons (April–July and October–November) (Goula Bi Tie et al. 2007). In this region, soil samples were taken from two cocoa plantations in two localities, namely, Amanikro (AKO) and Dahopa-Ourépa (DHP). In each plantation, three soil samples were taken with an auger to a 20 cm depth. A total of 48 samples were taken, 3 replicates per plantation, in 2 plantations of 8 localities.

Each soil sample comprised 12 sub-samples taken from a radius of 3 m (4 subsamples) and 6 m (8 subsamples) from a central point. Weeding had been regularly performed around the trees such that the soil samples always were taken from an area where there was no ground cover vegetation. After homogenization of the subsamples, 300 g was removed and packed in plastic bags. Samples were kept at 4 °C and then were sent to the University of Lausanne (Switzerland) 2 weeks after collection. Upon arrival, samples were conserved at − 20 °C until DNA was extracted.

Based on GPS coordinates, climatic data for the locations was obtained by rasterizing and stacking the layers of 19 bioclimatic variables available from the CHELSA (climatologies at high resolution for the Earth’s land surface areas) database (Karger et al. 2017). The values from the CHELSA database for each plantation in the eight localities are presented in Table S1.

### Determination of soil chemical properties

Soil pH was measured in a 1:1 soil/water solution. Organic carbon and organic matter (organic matter = 1.725 × % organic C) were determined by titration using the Walkley‐Black wet digestion method. Exchangeable cations in soil (K, Ca, and Mg) were measured by displacing cations with an EDTA-buffered solution of ammonium acetate at pH = 4.8; then, the concentration of each element was measured using a flame photometer. Organic nitrogen (N) was determined using the Kjeldahl method. Available Olsen’s extractable phosphorus (as H_2_PO_4_) was determined using the molybdenum blue reaction and measuring absorbance at 660 nm. The values of the chemical variables measured in the soil are presented in Table S2.

### DNA extraction, amplification, and sequencing

Genomic DNA was extracted from 250 mg of each soil sample using the PowerSoil DNA Isolation Kit (Qiagen, Hilden, Germany). After extraction, DNA was quantified using a Quantus™ Fluorometer (Promega).

A nested PCR targeting a portion of the small subunit (SSU) rRNA gene was performed using AML1-AML2 primers (Lee et al. 2008) followed by NS31 and AM1 primers (Öpik et al. 2008). PCR products were approximately 550 bp for NS31-AM1 and 800 bp for AML1–AML2. Primers AML1-AML2 were used because of their high specificity to AMF sequences and mismatching to non-AMF sequences (i.e., avoiding amplification of non-AMF DNA). Then, the NS31 and AM1 pair was used to obtain a shortened fragment that is suitable for high throughput sequencing (HTS) and includes a highly polymorphic region that is phylogenetically informative for AMF OTU determination (Öpik et al. 2013). Most published data on AMF community diversity have been obtained using the region amplified by the primers NS31 and AM1. This allowed us to make a direct comparison of our data with the extensive database of published sequences obtained using these primers, and also offers the possibility of a robust phylogenetic placement of AMF sequences (Öpik et al. 2010).

The first PCR was set up with a Taq PCR Core Kit (Qiagen, Hilden, Germany) using 1× coral load PCR buffer, 0.5× Q-solution, 2.5 mM MgCl_2_, 0.25 mM dNTP mix, 0.1 U/μL Taq DNA polymerase, 0.1 mM of each primer, and 25 ng of DNA in a volume of 25 μL. Thirty amplification cycles were carried out with denaturation at 94 °C for 60 s, hybridization at 50 °C for 60 s, and elongation at 72 °C for 60 s. Successful amplifications, verified by gel electrophoresis, were purified using AMPure® beads (Beckman Coulter Genomics) and diluted to a concentration of 1 ng/μL before performing the second reaction. The second amplification was the same as the first but in a reaction volume of 50 μL following the same steps and the same number of amplification cycles as the first reaction. Importantly, the NS31 primer contained a five-base barcode for library multiplexing.

Library preparation was performed with the TruSeq™ Sample Preparation Kit but omitting the DNA fragmentation step. Quality of the resulting libraries was assessed with a Fragment Analyzer™ and quantified with Qbit. The libraries were then sequenced using the Illumina MiSeq 2 × 300 bp paired-end run protocol.

### Sequence processing and bioinformatic analyses

Raw data were checked with FastQC v0.11.4. After library quality check, the adaptor sequences were trimmed using TagCleaner (Schmieder et al. 2010). Reads were then quality-filtered (min_qual_mean 25) and trimmed using Prinseq-lite version 0.20.4 (Schmieder and Edwards 2011). Low-quality 3′-ends were trimmed and reads containing uncalled bases (N) removed. Reads were then demultiplexed according to exact barcode sequences, and paired reads were merged using PEAR (Zhang et al. 2014) by specifying a minimum overlapping region of 20 bp. Primer and barcode sequences were then removed and reads below 500 bp were discarded. A summary of the sequence processing is found in Table S3. At this stage, remaining reads were trimmed to a 500 bp length and sorted according to their nucleotide sequence (hereafter referred as rRNA allele) and relative abundance in the dataset. Alleles that were found only in one sample were discarded from the dataset.

### OTU model selection

We followed the OTU model selection process proposed by Ordoñez et al. (2020). The pipeline for selecting the optimal model for clustering sequences into OTUs is described in detail in Supplementary note 1 of Ordoñez et al. (2020). Briefly, the whole catalog of alleles, weighted by their occurrence in the dataset, was subjected to de novo OTU-picking using DBC454 v1.4.5 (Pagni et al. 2013). The selection of the clustering algorithm parameters was done in an iterative process aiming for OTU taxonomic coherence (i.e., selected models had no OTUs composed of a mixture of AMF and non-AMF alleles). We used BLAST results from each rRNA allele against the INSD/EMBL (http://www.insdc.org) and MaarJAM (https://maarjam.botany.ut.ee) databases to calibrate the dissimilarity cutoff distances in the software. Among the selected clustering models, we chose the one harboring the highest number of OTUs in order to not underestimate the diversity in a biome where documented characterizations of AMF communities are scarce. Selected model parameters for the DBC454 algorithm were -n 50 (minimum cluster size), -d 20 (initial cutoff distance), and -e 46 (final cutoff distance). Sampling intensity for each plot was assessed using the deviance between estimated species richness obtained with a replicate-based Chao1 index and the observed OTU richness. Consensus sequences for each OTU, obtained from the clustering model, were built based on nucleotide frequencies at each position in the SSU sequences.

### Statistical analyses

All statistical analyses were performed using R v3.6.0 (R Core Team 2019). In order to assess the degree of overlap of climatic variables in the localities, a PCA was performed on the standardized Euclidean distances of the environmental data (CHELSA and soil variables). To obtain an indication of the individual contribution of each of these two groups of variables, PCAs were built using CHELSA and soil data separately. Analysis of similarity (ANOSIM) was used to test the dissimilarity between groups. An *R* value close to 1 suggests dissimilarity between groups while an *R* value close to 0 suggests an even distribution of high and low ranks within and between groups.

Following OTU model selection, taxa abundances were recorded in a matrix. The non-parametric Kruskal–Wallis test was applied to compare regions and localities. Phyloseq (McMurdie and Holmes 2013) was used to estimate richness and alpha diversity indices. As alpha diversity indices differentially weigh the importance of dominant and rare species, multiple indices were considered to avoid index-dependent interpretations. The non-parametric Dunn’s multiple comparison test and Kruskal–Wallis test were applied to identify differences between regions and localities.

To explore the relationship between community turnover and geographic location, distances between sampling points were calculated from GPS coordinates using a geodesic approximation (WGS84) as implemented in the R package geosphere (Hijmans 2019). The link between community dissimilarity (beta diversity) and environmental data was analyzed with a canonical ordination (redundancy analysis) built with the Bray–Curtis dissimilarity index (on Hellinger transformed abundance data) using the R package ‘vegan’ (Oksanen et al. 2019) with the climatic data from CHELSA and the measured soil variables as explanatory factors. RDA is a method combining regression and PCA in which multiple linear regressions are performed followed by a PCA on the fitted values. This method looks for a series of linear combinations of the explanatory variables (climate and soil in this study) that best explain the variation of the response matrix (beta diversity in this study). Variance partitioning was calculated from the RDA using vegan’s function varpart. Variance partitioning intends to quantify the variation explained by different subsets of the variables (e.g., soil and climate) when controlling for the effect of the other subsets.

Consensus sequences from the OTU selection model were aligned using MAFFT 7.058 (Katoh and Standley 2013) and a phylogenetic tree was constructed with RAxML (Stamatakis 2014) using a GTR + G nucleotide substitution model with the SSU rRNA sequence of *Schizosaccharomyces pombe* SSU as the tree root. The tree was selected based on maximum likelihood (ML) score and node probability was assessed using bootstrap resampling from the ML tree. This tree was used to calculate phylogenetic alpha diversity via mean pairwise distances (MPD) using the R package ‘picante’ (Kembel et al. 2010). The importance of this metric reflects that if two communities have the same number of equally abundant species, they will have the same alpha diversity. However, if these species all belong to a same clade, their phylogenetic (and potentially functional) diversity is low. MPD measures branch lengths to obtain an indication on how phylogenetically diverse a community is within an assemblage (Tucker et al. 2017). Thus, a community with high phylogenetic diversity will comprise less related AMF taxa (which in turn may be more functionally diverse) than a community with a low phylogenetic diversity.

## Results

**Fig. 1:**
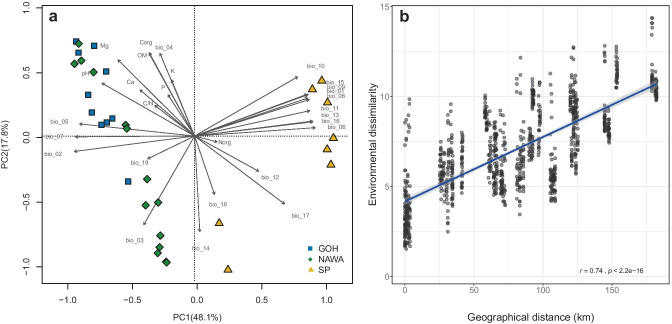
**a** Principal component analysis of the environmental data for all localities and regions (Gôh (blue), Nawa (green), and San Pedro (SP; yellow). Data include the 19 CHELSA variables (bio1 to bio19) (description of bio variables in Table S1) and the soil variables pH, organic carbon (Corg), and organic matter (OM), phosphorus (P), extractable potassium (K), magnesium (Mg), calcium (Ca), organic nitrogen (Norg), and carbon–nitrogen ratio (C/N) (Table S2). **b** Correlation of the Euclidean distance of the scaled environmental variables (CHELSA and soil) and the geographical distances between samples. All points are shown as gray dots; darker dots reflect dot overlaps. Gray shading around the blue line corresponds to the 95% confidence interval for predictions from the linear model

The PCA performed on the climatic and soil variables (Fig. 1a), along with permutational tests, showed an overlap of the environmental conditions in the Gôh and Nawa regions (ANOVA *p* = 0.068; ANOSIM R: 0.27, significance: 0.002) while the San Pedro region showed distinct environmental conditions (San Pedro–Gôh ANOVA *p* = 0.001; ANOSIM R: 0.66, significance: 0.001. San Pedro–Nawa ANOVA *p* = 0.001; ANOSIM R: 0.78, significance: 0.001). When PCAs were calculated using the CHELSA and soil data independently, the CHELSA space revealed a similar difference between regions (ANOVA *p* = 0.001; ANOSIM R: 0.66, significance: 1e-04) while the soil variables did not reveal significant differences (ANOVA *p* = 0.277; ANOSIM R: 0.16, significance: 6e-04) (Fig. S2). There was a significant correlation between environmental and geographic distances showing that distant geographic locations were different in their climate and soil chemistry (Fig. 1b).

**Fig. 2:**
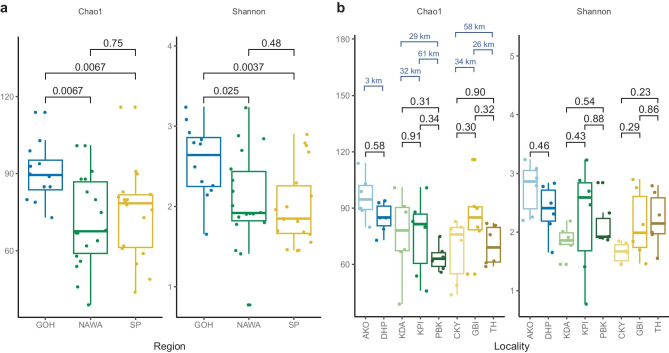
Richness (Chao1) and alpha diversity (Shannon) comparisons by region **a** and locality **b** at the OTU level. Non-parametric Wilcoxon **a** and Dunn’s **b** tests were used to compare means with the probabilities of differences between pairs of means indicated above brackets. **b** (Chao1) The geographic distances between localities within each region in blue above brackets. Points are displaced horizontally to improve visibility. Region designations and colors are as in Fig. 1; locality codes are presented in Methods: Sampling sites. Upper and lower whiskers of the boxplots extend from the hinge to the largest or smallest value at the most 1.5*IQR

Deviance between observed OTU richness and replicate-based Chao1 index was not significant, and rarefaction curves of individual samples reached the plateau phase (data not shown), both indicating that sufficient sampling was achieved. Comparisons of OTU alpha diversity between regions (Fig. 2a) showed that AMF communities in Gôh tended to be richer and more diverse than those in the two other regions. The dominant families across all the localities were the *Glomeraceae* and *Acaulosporaceae* (Fig. S4a). The Kruskal–Wallis test, comparing family abundance between regions, revealed *Archaeosporaceae*, *Ambisporaceae*, *Acaulosporaceae*, *Geosiphonaceae*, and *Pacisporaceae* to be differentially abundant between regions (FDR corrected *p* < 0.05). Likewise, pairwise comparisons, using a Wilcoxon test, showed that abundance of all of the above-mentioned families was lower in Gôh than in the other two regions (Fig. S4b).

Variation in OTU alpha diversity tended to be independent of geographical distance. For example, Shannon alpha diversity did not differ neither between localities 61 km apart (KPI and PBK) nor for those localities separated by 29 km (KDA and PBK) (Fig. 2b). Likewise, phylogenetic alpha diversity differed between localities KDA and PBK separated by 29 km, but did not differ between other localities that were as much as 61 km apart (Fig. S5).

Alpha diversity indices (Shannon Fig. 2, Fisher, Simpson Fig. S3) were correlated with some of the climatic and soil gradients observed across the study area. Alpha diversity was strongly positively correlated with soil pH and negatively correlated with annual precipitation (Fig. S6a, b). pH largely separated the alpha diversity of localities CKY, GBI, TH, and KDA, which have a mean pH lower than 5.7, from the localities AKO, DHP, KPI, and PBK, having a mean pH higher than 6.9. The annual precipitation grouped localities into those with a mean value lower than 1330 mm/year (AKO, PBK, and DHP) and a mean value higher than 1430 mm/year (GBI, KDA, KPI, CKY, and TH). Alpha diversity was negatively correlated with mean annual air temperature (Fig. S6c) and also negatively correlated with soil Mg concentration (Fig. S6d). The pH, annual precipitation, mean annual air temperature, and Mg concentration gradients, however, did not follow the geographic locations of the sampling points. For example, pH grouped KDA in the Nawa region (Fig. S1) with samples from the San Pedro region to the south (Fig. S6a), and in the case of mean annual air temperature, TH in the San Pedro region (a southern sampling location) grouped with DHP in the Gôh region (the northernmost sampling location).

The abundance of some taxa differed significantly among communities and correlated with some climatic and edaphic variables. Among the variables found to be most related to the abundance of these OTUs were pH, Mg, mean annual air temperature, annual precipitation, mean daily air temperature of the wettest quarter, precipitation of the wettest month, and mean monthly precipitation of the wettest quarter (Table S4).

**Fig. 3:**
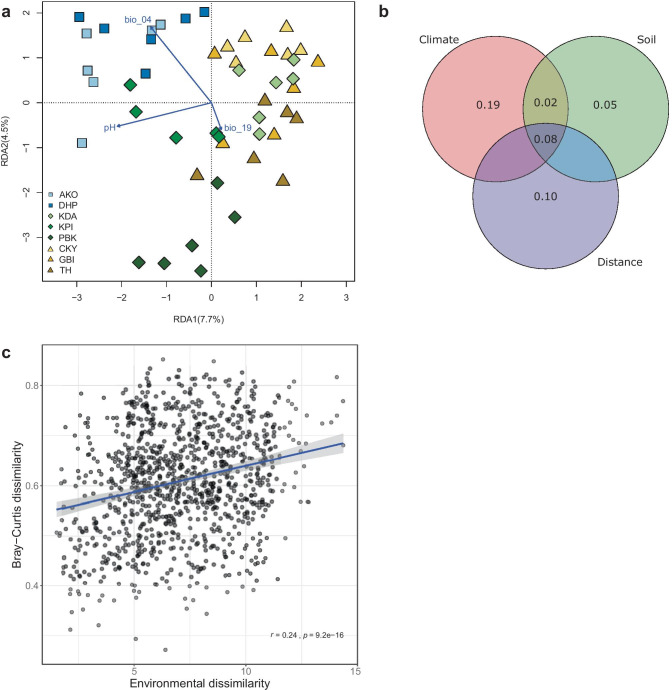
Beta diversity analyses by locality and environment. Sampling points are colored by region, Gôh (blue), Nawa (green), and San Pedro (yellow). **a** Redundancy analysis of beta diversity at the different localities. The OTU abundance was Hellinger transformed prior to the RDA. The standardized explanatory matrix included the 19 bio variables from the CHELSA database, and the soil variables measured at each sampling point for which a forward selection procedure was applied. Retained variables are shown. Loadings were omitted for clarity. **b** Variance partitioning of the Bray–Curtis dissimilarity (db-RDA) at the sampling points (beta diversity). The standardized explanatory matrices included: (a) the 19 bio variables from the CHELSA database (climate), (b) the soil variables measured for the sampling points (soil), and (c) a matrix of geographical distances calculated from the GPS coordinates (distance). The figures correspond to the proportion of variation in community beta diversity accounted for by each one of the explanatory matrices and their combined effects. Values lower than 0.01 are not shown. Negative values can occur because of adjustments in the model. They are interpreted as zeros because they correspond to cases where the explanatory variables explain less variation than random normal variables. **c** Correlation of the Euclidean distance of the scaled environmental variables (CHELSA and soil) and the Bray–Curtis dissimilarities between samples

The redundancy analysis (integrating soil and climate variables) showed beta diversity (i.e., community composition differences) among samples (Fig. 3a; Permanova *F* = 1.3878, *p* = 0.001), with the first two axes explaining 14.1% of the total variation. However, differences in AMF community composition in localities were not separated by region, so the distribution of the samples did not match the environmental overlap (Fig. 3a versus Fig. 1). The variance partitioning analysis using three explanatory matrices (CHELSA climatic data, soil variables, and a matrix of the geographic distances) showed that separately the climatic data explained 19% of the observed variance in AMF beta diversity, the geographic distance 10%, and the soil chemical variables 5% (Fig. 3b). Sixty-six percent of the variance remained unexplained. There was a significant correlation (although relatively weak) between environmental distance (Euclidean distances of standardized soil and climate data) and the pairwise Bray–Curtis dissimilarity index with increasing environmental differences between samples correlated with increasing differences in AMF community composition (Fig. 3c).

**Fig. 4:**
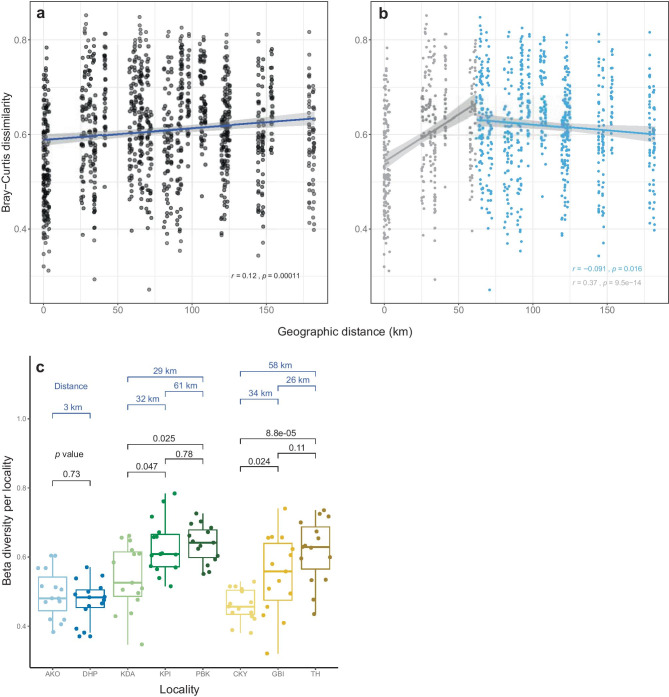
Bray–Curtis dissimilarity index (beta diversity) plotted against a matrix of geographical distances between sampling points calculated from the GPS coordinates **a** and divided into two groups at 62 km distance in **b** which was the largest distance separating localities within the same region. **c** Beta diversity per locality (as Bray–Curtis). Dunn’s test was used to make the between-locality comparisons and the corresponding probabilities are shown. Geographical distances between localities within regions are shown in blue. Points are displaced horizontally to improve legibility

The Bray–Curtis dissimilarity index plotted against the geographical distance matrix showed an overall trend in which beta diversity (AMF community compositional differences) increased with the distance between samples (Fig. 4a). However, when separating the data into comparisons between samples situated within the same region (sampling points with a ≤ 62 km distance between them), there was a change in the trend of dissimilarity of the AMF community compositions from positive to slightly negative (Fig. 4b). Within the same region, at comparable distances, some between-locality comparisons showed statistically significant and relatively large differences in AMF community composition while others did not (Fig. 4c).

## Discussion

By studying the AMF distribution across different geographical scales, we found that AMF composition and diversity in Côte d’Ivoire cocoa plantations were not strictly linked to the distance between sampling points but were most related to the climatic differences among the localities. This conclusion derives from OTU alpha diversity in closely situated localities sometimes differing while OTU alpha diversity from localities that were distant was similar (Fig. 2). Likewise, increasing AMF community dissimilarity (beta diversity) with geographic distance (expected in most cases) was only observed among samples within a 62 km range. AMF community dissimilarity tended to slightly decrease with distance beyond 62 km, implying that communities increase in resemblance with increasing distance between them (Fig. 4b). It is difficult to interpret the ecological significance of the magnitude of change in dissimilarity of the AMF communities with distance. This is because there are few studies in similar regions with which to compare. Second, even a small change in community composition can potentially have a large effect ecologically, if a key taxon is affected. Because we do not know the ecological roles of all different taxa, this is not possible to interpret. Given the strong environmental gradient in the study area (Fig. 1), the trend in dissimilarity decrease with distance suggests the possible existence of a homogenization process (environmental filtering), which is likely independent of climatic variables and distance, as the variance partitioning revealed.

The low AMF endemism reported by Davison et al. (2015) suggests that AMF biogeography is largely determined by local environmental conditions, and this agrees with the data presented here. However, under similar environmental conditions, and through interactions with the same species pool (Davison et al. 2016; Vályi et al. 2016), local communities are expected to converge upon a common composition and beta diversity should be low. Here, we found that samples within the same locality, thus with similar environmental conditions, showed large variation in terms of community composition measured as beta diversity (Fig. 4c). It previously has been reported that AMF assemblages within similar environments were largely unpredictable and presented a higher degree of stochasticity than models (environmental filtering, niche-based assembly, neutrality) would predict (Powell and Bennett 2016). The data presented here support this. However, most other studies originate from temperate areas and few data exist from tropical soils with which we can compare our data. Furthermore, there is a scarcity of studies of AMF community diversity at different spatial scales.

By sampling soil beneath cocoa trees where there was no other ground cover, we partially controlled for one factor (namely, plant diversity) that could influence AMF community composition. However, as in any other agroecosystem, there was a plant community that existed in the near vicinity of the cocoa trees that could potentially indirectly act as an additional environmental filter. However, it has been suggested that AMF diversity will not increase with an increase in plant species diversity once certain threshold of plant diversity is reached (Oehl et al. 2010). A study in Brazil, based on spore morphology, reported values of Shannon alpha diversity between 3.3 and 4.6 in natural and anthropic environments. As spore extraction captures only a fraction of the total community, this highlights the potential for additional diversity of AMF in tropical areas yet to be found. A work carried out in Cameroon, comparing natural and anthropic ecosystems, reported AMF spore diversity values (Shannon index) as low as 0.39 for agricultural sites, with forests being richer (0.49) (Snoeck et al. 2010). These figures are especially low considering reports of AMF spore diversity (Shannon) of 1.4 and 2.27 in alfalfa and sorghum crops in Sudan (Abdelhalim et al. 2014), 1.96 in maize in Brazil (de Mello et al. 2018), 2.65 in cassava (Sarr et al. 2019), and between 1.65 and 2.75 in this study using amplicon sequencing.

Clearly, spatio-temporal relationships need to be thoroughly studied (Bittebiere et al. 2020). However, neither the study by Kiers et al. (2011) or that of Zobel and Öpik (2014) have looked at tropical soils where plants are severely limited by low soil nutrient availability.

In this study, measurements of AMF alpha and beta diversity showed that results at one scale (e.g., the local level) do not reliably predict what alpha and beta diversity will be observed at another scale (e.g., region). The amount of variation explained by soil, climatic, and geographic variables still leaves most of the variance to be explained. Neutral or stochastic processes might contribute at small scales (Maherali and Klironomos 2012; Davison et al. 2016). The possible stochasticity may be the result of several extrinsic factors (e.g., anthropogenic intervention) and intrinsic factors (e.g., coexistence of different AMF taxa within the same plant) determining AMF community structure (Vályi et al. 2016). Importantly, observed variation could also be deterministic but attributed to variables not measured in this study.

Multiple studies have highlighted the importance of soil characteristics in regulating AMF species composition. pH and soil type often arise as stronger factors in determining the occurrence of AMF species than plant species diversity (Oehl et al. 2010; Boeraeve et al. 2019). Relationships between soil Mg concentrations and AMF community composition have rarely been reported. Here, pH indeed arose as a significant factor, not only influencing overall AMF alpha diversity and differential abundance of specific taxa and families of the Glomeromycotina, but also exhibiting large differences between regions. Potentially, a pH-mediated differential availability of nutrients to host plants may have caused a decrease in the amount of photosynthates allocated to mycorrhizal partners, which in turn, ultimately may have reduced AMF diversity. However, we lack the data to formally test this claim. This possible differential availability of nutrients also may have been influenced by climatic variables such as higher precipitation as seen in Fig. S6b (which may lead to higher runoff and leaching) and higher temperature (Fig. S6c) perhaps exacerbating weathering.

It has been reported that AMF community composition differs between annual and perennial plants (López-García et al. 2017). The higher relative abundance of *Glomeraceae* versus lower relative abundance of *Paraglomeraceae*, *Acaulosporaceae*, and *Diversisporaceae* (e.g., Séry et al. 2018) also may be related to certain phylogenetically conserved traits of AM fungal taxa (Maherali and Klironomos 2007). Boeraeve et al. (2019) reported that competition does not appear to play an important role in AMF community structure in herbs. If extrapolation of these results to longer-lived roots of cacao trees is considered, additional explanations of the prevalence of *Glomeraceae* may be the high number of hyphal fusions and anastomosis in this family which has been suggested as a trait conferring adaptation to soil disturbance (Oehl et al. 2010) typical of agroecosystems. Additionally, it is not unusual for AMF assemblages to exhibit highly prevalent “core” species found in most communities, and less prevalent “satellite” species found in few communities (Öpik et al. 2013). Here, Fig. S4 shows a dominance of Glomeraceae across the different regions, and the dominant OTUs all belonged to the Glomeraceae.

In summary, we conclude that AMF communities in cocoa plantations in Côte d’Ivoire are shaped by abiotic conditions, mainly climatic variables. However, assemblages and diversity metrics at the local scale did not reliably predict those at regional scales. The amount of variation explained by soil, climate and geography variables still leaves most of the variance to likely be explained by random processes, anthropogenic intervention, or possibly plant community composition. Additionally, seasonal variation in AMF abundance may have contributed to the unexplained variance in AMF community composition (Boeraeve et al. 2019).

The specific abiotic conditions of an agroecosystem can be changed considerably through management practices, which will in turn likely affect AMF community composition (Bainard et al. 2014; Moora et al. 2014). It is important to understand how these practices influence AMF and how AMF use can be implemented in cocoa plantations in Côte d’Ivoire. Likewise, it will be important to establish adequate management practices, for example revision of fertilizer applications (N’Guessan et al. 2017) to promote a functionally beneficial AMF community. Gaining understanding of processes involved in shaping tropical AMF communities and AMF establishment (Köhl et al. 2016) is much needed and could allow for the development of more sustainable and productive cocoa agroecosystems than at present.

## Supplementary information

Below is the link to the electronic supplementary material.

Supplementary file1 (DOCX 6193 KB)

## Data Availability

The sequence reads after quality control (see Materials and methods), soil variables data, and GPS coordinates of the sampling points were deposited at the European Nucleotide Archive and are available under the study accession PRJEB37876.
