# Supplementary material for: Hierarchical spatial sampling reveals factors influencing arbuscular mycorrhizal fungus diversity in Côte d’Ivoire cocoa plantations
Source: Mycorrhiza. 2021 Feb 27;31(3):289–300. doi: 10.1007/s00572-020-01019-w (PMC8068719; doi:10.1007/s00572-020-01019-w)
Supplement: Supplementary file 1 — Supplementary file1 (DOCX 6193 KB) [file 572_2020_1019_MOESM1_ESM.docx]

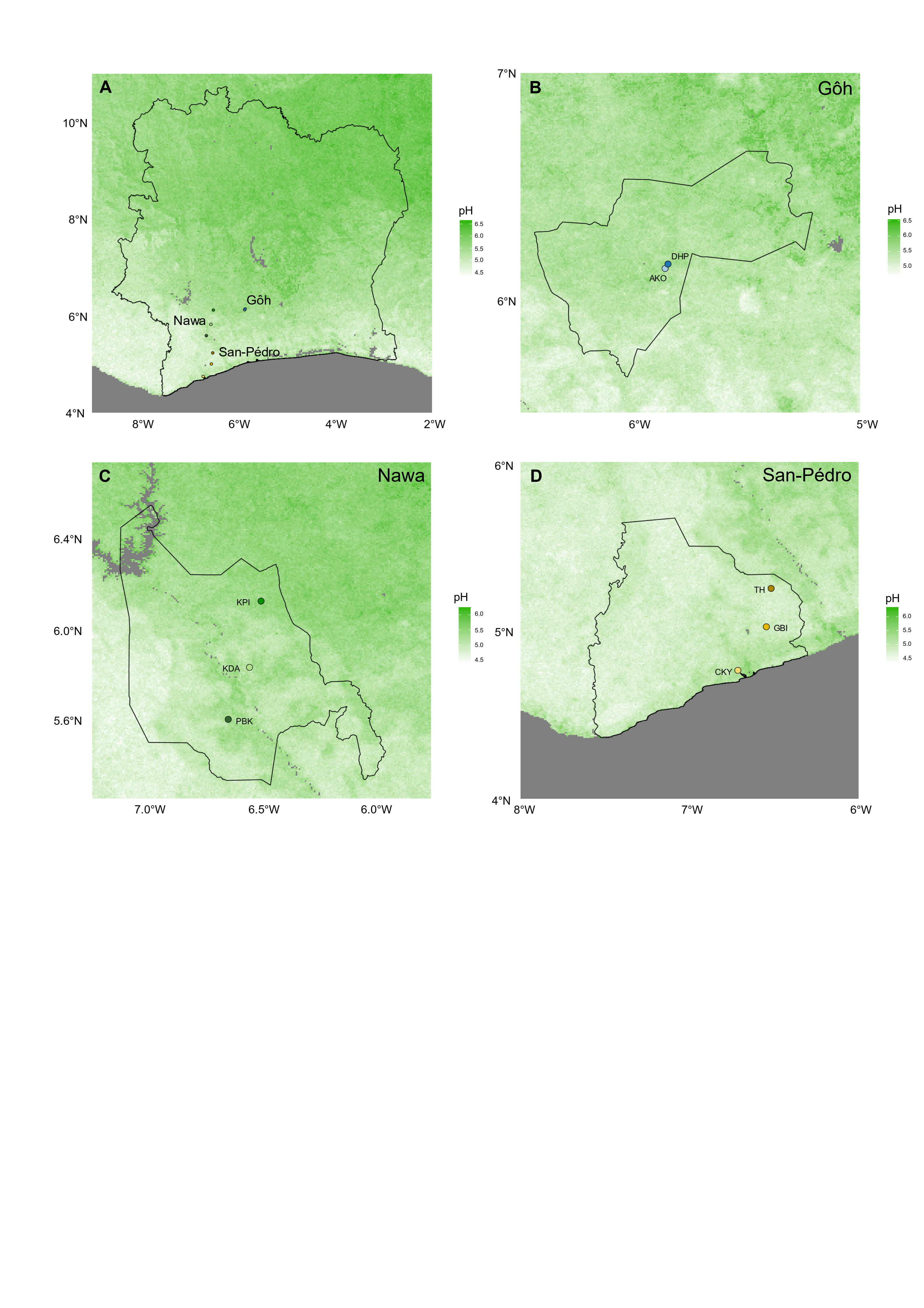


**Figure S1**. Geographical location of the sampled localities. Panel A shows the regions in the Ivory Coast; panels B to D the locations of the different localities within each of the three regions Gôh, Nawa and San-Pédro, respectively. The background shows the soil pH from the SoilGrids database (Hengl *et al.*, 2017). Gray represents water bodies (Atlantic Ocean in panels A and D and “Lac de Buyo” in panel C)


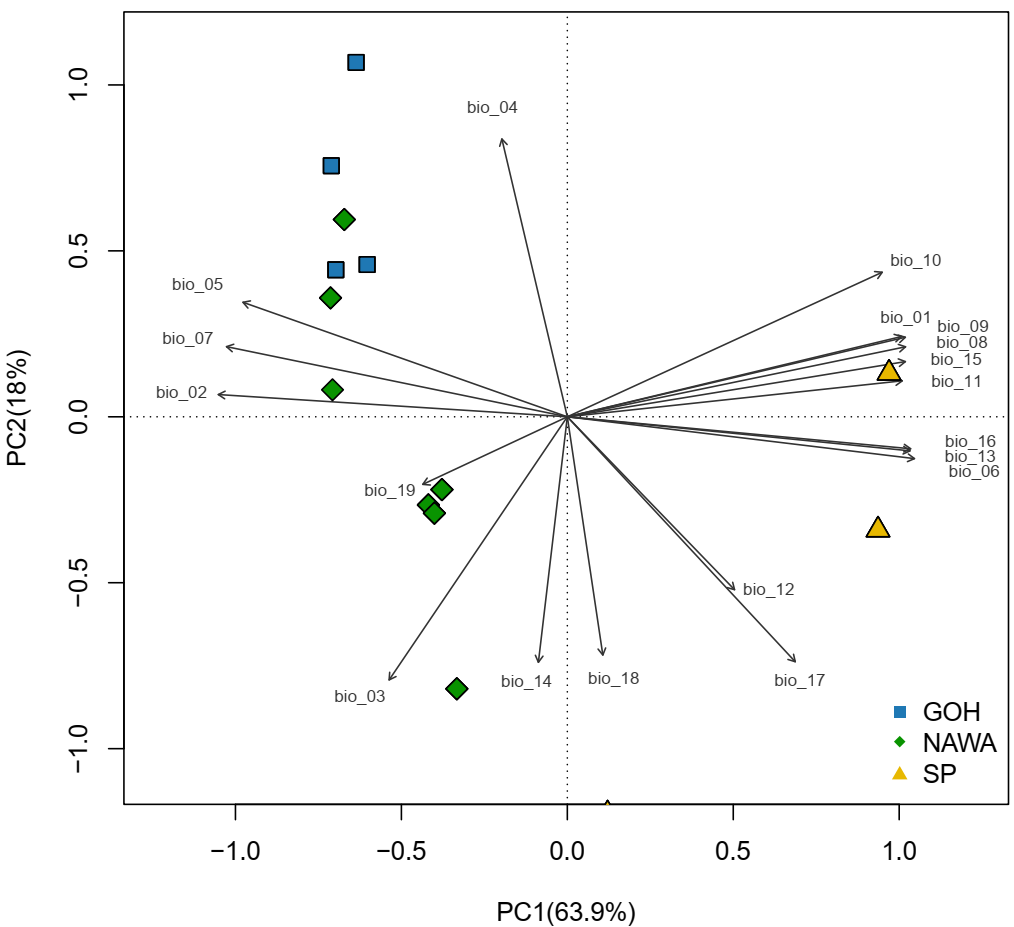


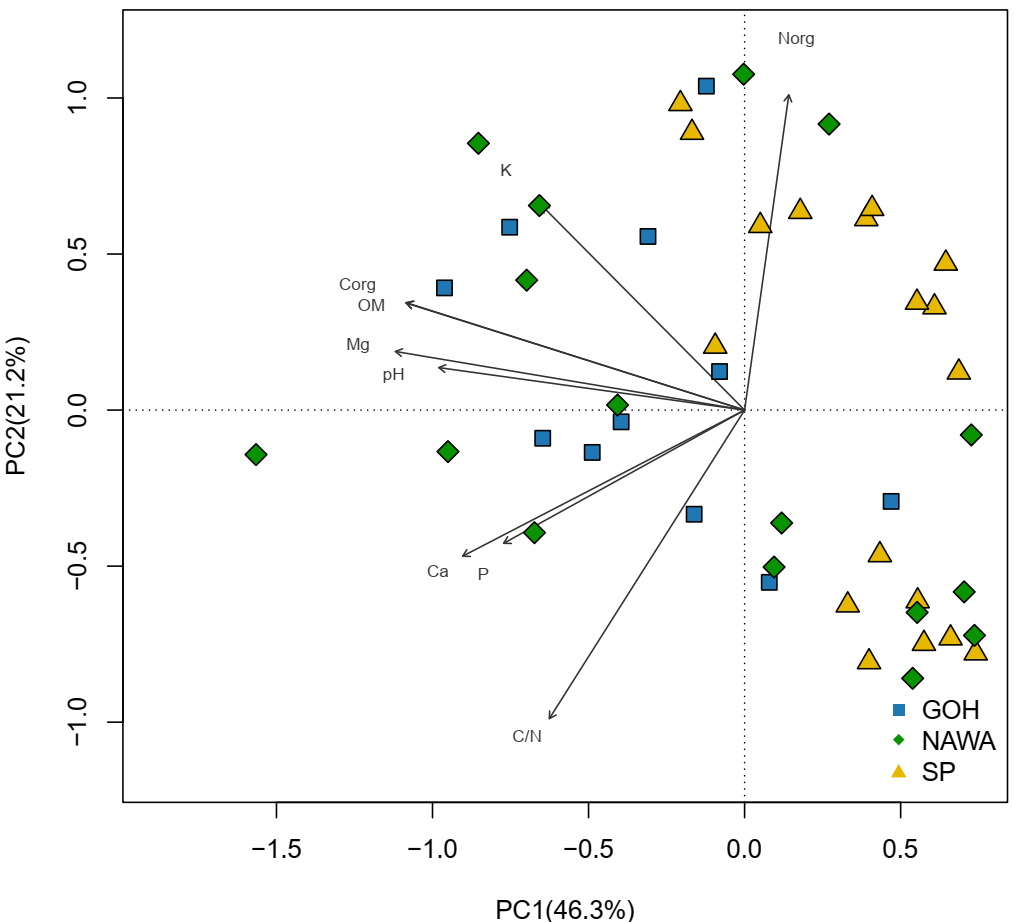


Figure S2. Principal component analysis of the environmental data at the different localities. Symbols reflect regions (Gôh (blue squares), Nawa (green diamonds) and San Pedro (SP; yellow triangles). The top panel includes the 19 CHELSA variables (bio1 to bio19) (description of bio variables is in Table S1) and the bottom panel the soil variables pH, organic carbon (Corg) and organic matter (OM), phosphorus (P), extractable potassium (K), magnesium (Mg) and calcium (Ca), organic nitrogen (Norg) and carbon-nitrogen ratio (C/N). Apparent lack of samples in the top panel corresponds to sites with highly similar conditions that are, thus, stacked on top of each other.


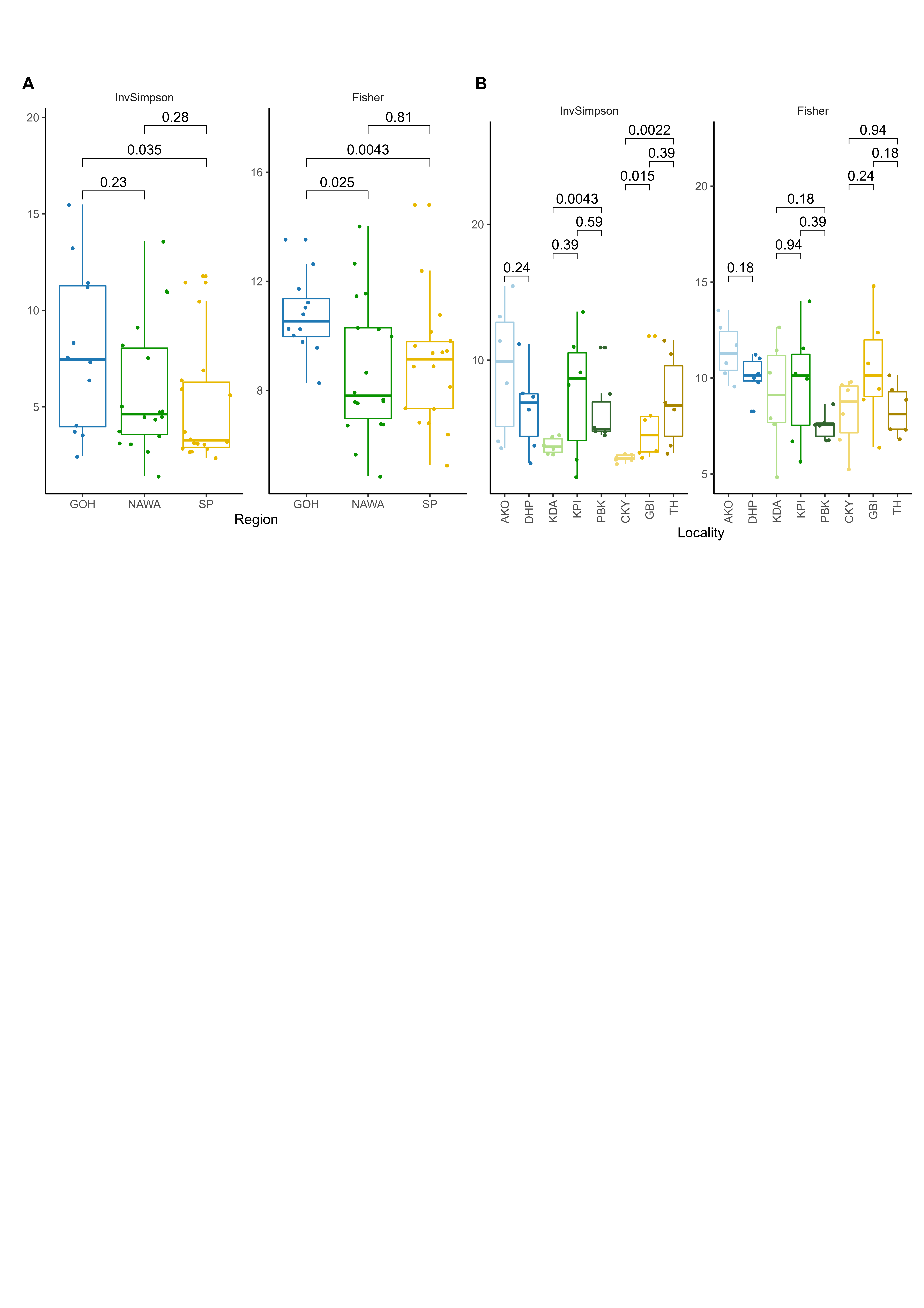


**Figure S3**. Alpha diversity comparisons by region (A) and locality (B) at the OTU level. Non-parametric Wilcoxon (A) and Dunn’s (B) tests were used to compare the means. Points were displaced horizontally to improve visibility. Region designations and colors are as in Figure S2; locality codes are presented in Methods: Sampling sites.

.


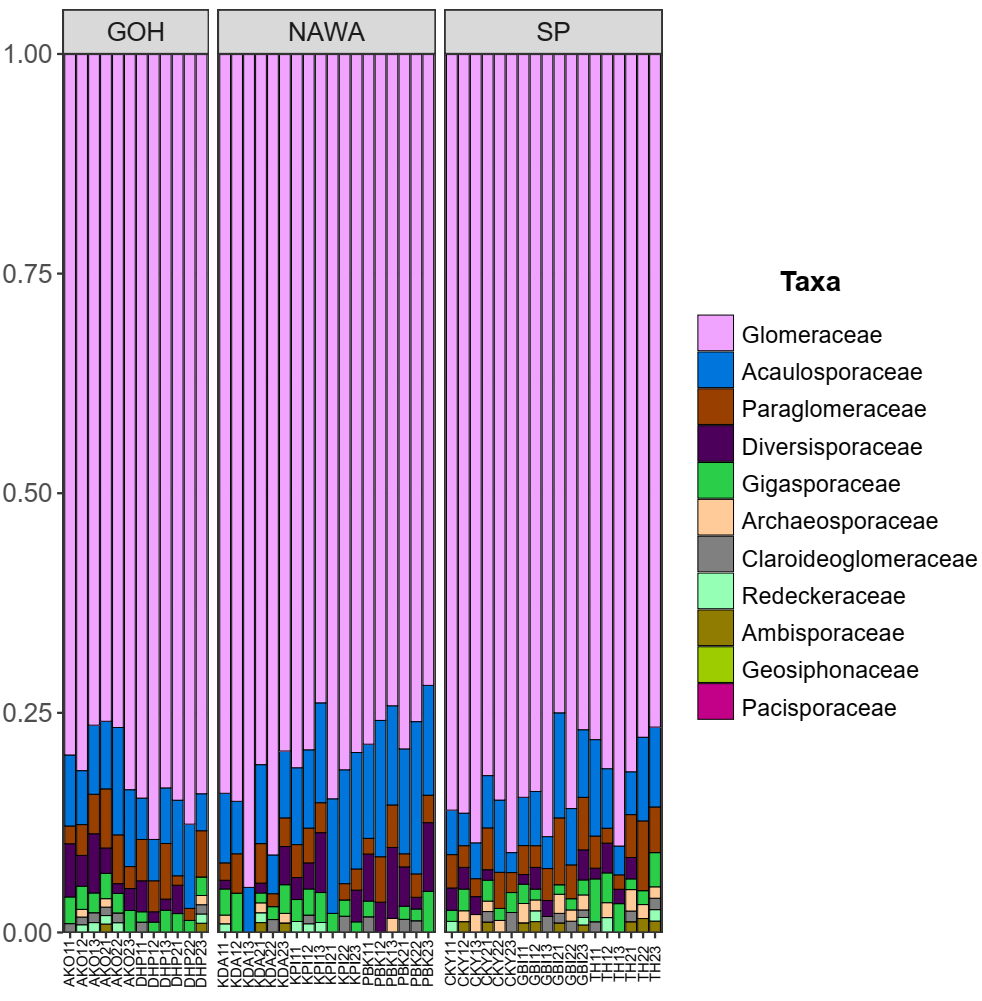


**A**

**B**


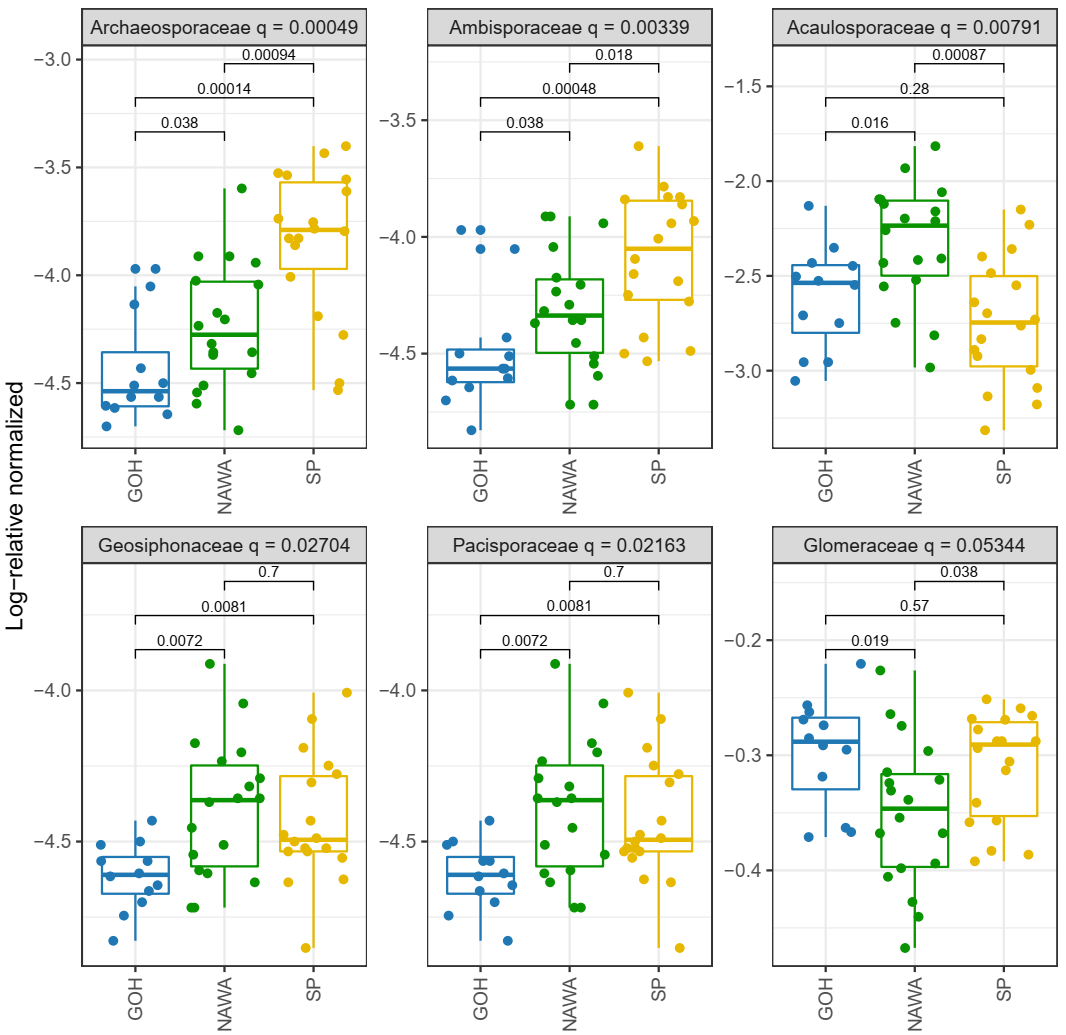


**Figure S4.** A) relative abundance of the Glomeromycotina families detected across the different regions and localities . B) Comparison of relative abundances (log-transform normalized ordinate axis) of families among regions. The q-value in the gray box shows the Kruskal-Wallis FDR corrected test for differences across regions. The significance value was set to 0.05, keeping Glomeraceae as a reference because it was the most abundant family. The pairwise comparisons between regions were performed with a Wilcoxon test. Points were displaced horizontally to improve visibility. Region designations and colors are as in Figure S2.


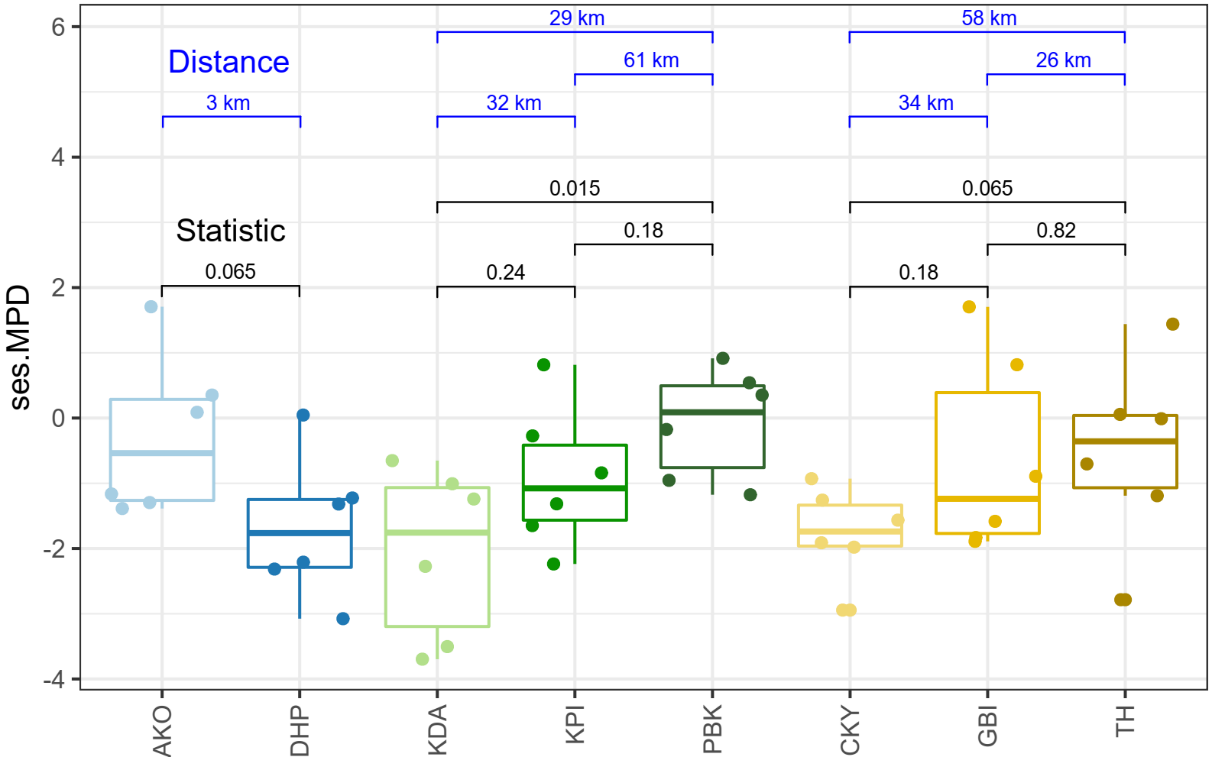


**Figure S5.** Phylogenetic alpha diversity as standardized effect size of mean pairwise distance (ses.MPD) across the localities. Geographical distances between localities of the same region are shown in blue and a Wilcoxon test comparing their ses.MPD in black. Points were displaced horizontally to improve visibility. Locality codes are presented in Methods: Sampling sites.


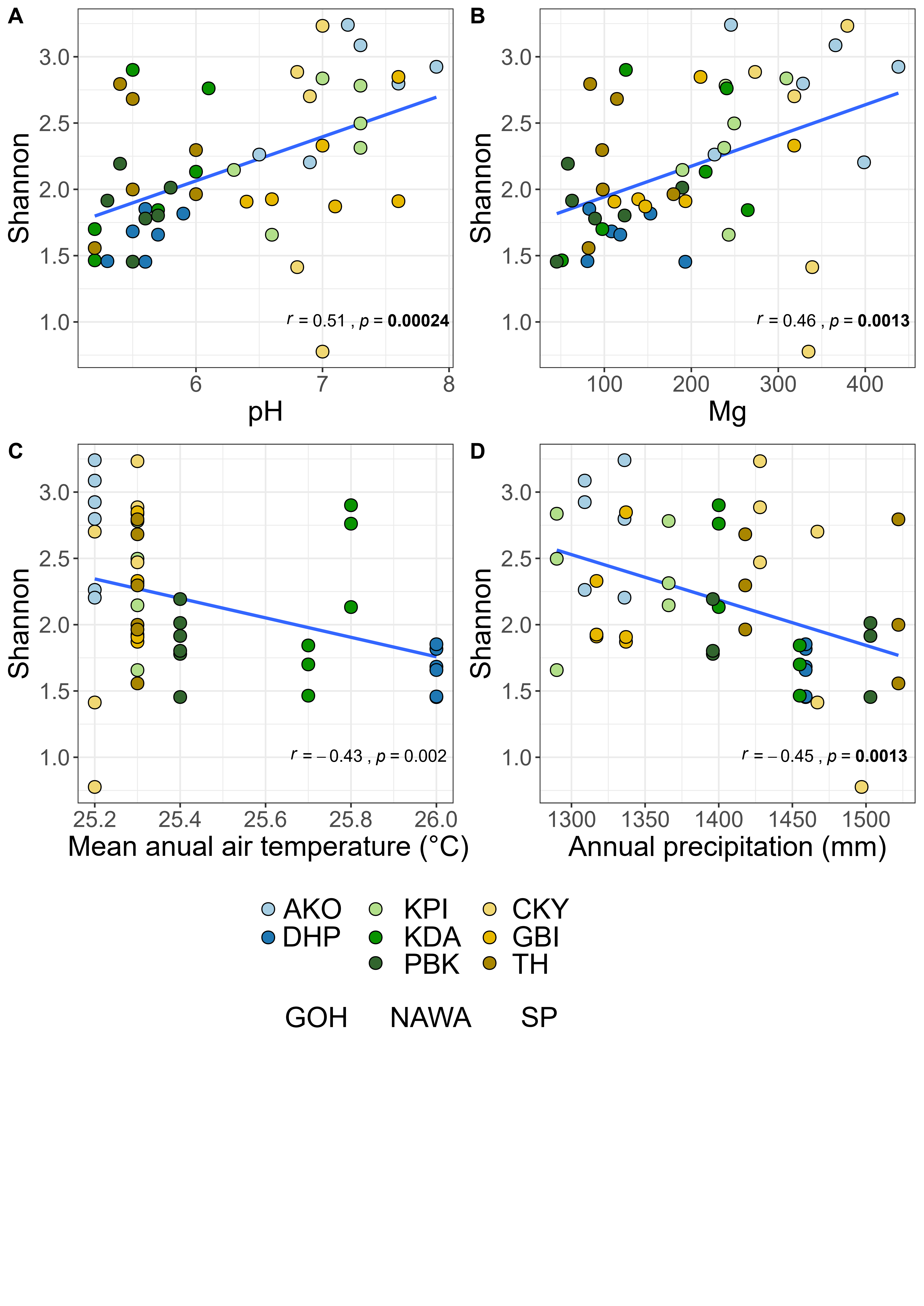


**Figure S6**. Correlation of environmental variables and Shannon alpha diversity index. The Spearman correlation coefficient and its significance are shown on each graph. Bold p-values indicate significant correlation after Bonferroni correction. These four variables were found to be significant using Simpson and Fisher alpha diversity indices (Bonferroni-corrected for multiple post hoc correlations). Soil variables (A and D) were measured at sampling points, and climatic data (B and D) were extracted from the CHELSA database (see Methods). Region designations and colors are as in Figure S2; locality codes are presented in Methods: Sampling sites.

**Table S1:** Climatic data extracted from the CHELSA database based on GPS coordinates averaged per plantation. Temperature related variables are expressed in C° times 10 and precipitation in kg m^-2^. **bio1:** mean annual air temperature; **bio2:** mean diurnal air temperature range; **bio3:** isothermality; **bio4:** temperature seasonality; **bio5:** mean daily maximum air temperature of the warmest month; **bio6:** mean daily minimum air temperature of the coldest month; **bio7:** annual range of air temperature; **bio8:** mean daily mean air temperatures of the wettest quarter; **bio9:** mean daily mean air temperatures of the driest quarter **bio10:** mean daily mean air temperatures of the warmest quarter; **bio11:** mean daily mean air temperatures of the coldest quarter; **bio12:** annual precipitation amount; **bio13:** precipitation amount of the wettest month; **bio14:** precipitation amount of the driest month; **bio15:** precipitation seasonality; **bio16:** mean monthly precipitation amount of the wettest quarter; **bio17:** mean monthly precipitation amount of the driest quarter; **bio18:** mean monthly precipitation amount of the warmest quarter; **bio19:** mean monthly precipitation amount of the warmest quarter. **Altitude:** mean altitude in meters above sea level. Locality codes are presented in Methods: Sampling sites.

| **Region** | **Locality** | **Plantation** | **bio1** | **bio2** | **bio3** | **bio4** | **bio5** | **bio6** | **bio7** | **bio8** | **bio9** | **bio10** | **bio11** | **bio12** | **bio13** | **bio14** | **bio15** | **bio16** | **bio17** | **bio18** | **bio19** | **Altitude** |
| --- | --- | --- | --- | --- | --- | --- | --- | --- | --- | --- | --- | --- | --- | --- | --- | --- | --- | --- | --- | --- | --- | --- |
| Gôh | AKO | 1 | 252 | 55 | 537 | 954 | 308 | 205 | 103 | 251 | 253 | 266 | 238 | 1309 | 195 | 25 | 48 | 560 | 87 | 314 | 241 | 222 |
|  | AKO | 2 | 252 | 55 | 537 | 950 | 308 | 205 | 102 | 251 | 253 | 265 | 238 | 1336 | 200 | 26 | 48 | 573 | 90 | 321 | 245 | 201 |
|  | DHP | 1 | 253 | 55 | 536 | 956 | 309 | 206 | 103 | 252 | 254 | 267 | 239 | 1290 | 192 | 24 | 48 | 550 | 85 | 311 | 241 | 224 |
|  | DHP | 2 | 253 | 55 | 536 | 957 | 308 | 206 | 103 | 252 | 254 | 266 | 238 | 1366 | 203 | 26 | 48 | 583 | 90 | 329 | 255 | 221 |
| Nawa | KDA | 1 | 254 | 52 | 549 | 883 | 305 | 210 | 95 | 253 | 255 | 266 | 241 | 1503 | 212 | 28 | 44 | 602 | 102 | 337 | 343 | 159 |
|  | KDA | 2 | 254 | 52 | 548 | 885 | 305 | 210 | 95 | 253 | 255 | 266 | 241 | 1396 | 195 | 27 | 44 | 555 | 98 | 312 | 317 | 141 |
|  | KPI | 1 | 253 | 55 | 537 | 919 | 308 | 206 | 102 | 252 | 254 | 266 | 239 | 1428 | 198 | 23 | 47 | 537 | 79 | 324 | 367 | 231 |
|  | KPI | 2 | 252 | 55 | 536 | 918 | 307 | 205 | 102 | 251 | 253 | 265 | 239 | 1477 | 204 | 23 | 47 | 557 | 81 | 337 | 378 | 225 |
|  | PBK | 1 | 253 | 49 | 561 | 859 | 301 | 213 | 88 | 253 | 255 | 265 | 241 | 1317 | 196 | 26 | 44 | 553 | 100 | 286 | 279 | 149 |
|  | PBK | 2 | 253 | 49 | 560 | 862 | 301 | 213 | 88 | 252 | 255 | 265 | 240 | 1337 | 199 | 26 | 44 | 562 | 101 | 289 | 282 | 142 |
| San Pedro | CKY | 1 | 260 | 27 | 484 | 920 | 291 | 235 | 56 | 260 | 269 | 273 | 247 | 1459 | 350 | 25 | 75 | 963 | 103 | 310 | 250 | 19 |
|  | CKY | 2 | 260 | 27 | 484 | 920 | 291 | 235 | 56 | 260 | 269 | 273 | 247 | 1459 | 350 | 25 | 75 | 963 | 103 | 310 | 250 | 12 |
|  | DHP | 1 | 257 | 35 | 547 | 895 | 293 | 229 | 64 | 257 | 261 | 270 | 244 | 1455 | 327 | 25 | 68 | 892 | 110 | 345 | 247 | 69 |
|  | DHP | 2 | 258 | 35 | 545 | 896 | 294 | 230 | 64 | 258 | 262 | 270 | 245 | 1400 | 321 | 24 | 69 | 873 | 106 | 327 | 236 | 54 |
|  | TH | 1 | 253 | 42 | 577 | 867 | 294 | 221 | 73 | 252 | 255 | 265 | 240 | 1522 | 291 | 28 | 55 | 804 | 116 | 395 | 276 | 132 |
|  | TH | 2 | 253 | 42 | 576 | 865 | 295 | 221 | 74 | 253 | 256 | 265 | 240 | 1418 | 270 | 26 | 55 | 746 | 109 | 367 | 256 | 126 |

**Table S2:** Soil properties measured at the sampling points and averaged per plantation. **OM**: organic matter (%);**P**: available phosphorus measured as Olsen extractable P (mg/Kg); **K, Mg, Ca**: extractable K, Mg, Ca (mg/Kg); **Norg**: organic nitrogen; **C/N**: carbon/nitrogen ratio; **Corg**: organic carbon (%). Each value represents the mean of three replicates per plantation. Locality codes are presented in Methods: Sampling sites.

| **Region** | **Locality** | **Plantation** | **pH** | **OM** | **P** | **K** | **Mg** | **Ca** | **Norg** | **C/N** | **Corg** |
| --- | --- | --- | --- | --- | --- | --- | --- | --- | --- | --- | --- |
| **Gôh** | AKO | 1 | 7.2 | 3.7 | 7.4 | 52.4 | 343.5 | 1872.0 | 0.09 | 30.6 | 2.12 |
|  | AKO | 2 | 7.2 | 4.0 | 3.9 | 56.1 | 324.2 | 1341.0 | 0.50 | 7.6 | 2.33 |
|  | DHP | 1 | 7.0 | 3.7 | 1.8 | 88.7 | 267.2 | 1175.3 | 0.21 | 9.9 | 2.13 |
|  | DHP | 2 | 7.0 | 2.7 | 1.8 | 50.4 | 222.3 | 873.7 | 0.55 | 8.3 | 1.57 |
| **Nawa** | KDA | 1 | 5.5 | 2.0 | 3.2 | 43.7 | 99.3 | 666.0 | 0.52 | 12.5 | 1.17 |
|  | KDA | 2 | 5.6 | 1.8 | 2.9 | 33.9 | 90.2 | 970.0 | 0.30 | 7.6 | 1.03 |
|  | KPI | 1 | 6.9 | 4.4 | 9.9 | 92.8 | 326.4 | 1557.5 | 0.21 | 12.1 | 2.50 |
|  | KPI | 2 | 6.9 | 4.7 | 2.5 | 79.7 | 330.7 | 1837.7 | 0.48 | 10.1 | 2.72 |
|  | PBK | 1 | 7.1 | 2.9 | 3.7 | 87.9 | 216.9 | 971.7 | 0.80 | 5.2 | 1.68 |
|  | PBK | 2 | 7.0 | 2.7 | 4.3 | 38.5 | 156.4 | 1344.3 | 0.50 | 8.7 | 1.57 |
| **San Pedro** | CKY | 1 | 5.7 | 2.3 | 3.0 | 37.8 | 114.7 | 857.7 | 0.35 | 7.8 | 1.35 |
|  | CKY | 2 | 5.5 | 3.9 | 4.0 | 73.2 | 130.7 | 771.0 | 1.00 | 2.3 | 2.23 |
|  | DHP | 1 | 7.0 | 3.7 | 1.8 | 88.7 | 267.2 | 1175.3 | 0.21 | 9.9 | 2.13 |
|  | DHP | 2 | 7.0 | 2.7 | 1.8 | 50.4 | 222.3 | 873.7 | 0.55 | 8.3 | 1.57 |
|  | TH | 1 | 5.4 | 2.2 | 3.9 | 33.4 | 87.9 | 922.7 | 0.65 | 4.4 | 1.28 |
|  | TH | 2 | 5.8 | 2.7 | 2.1 | 54.9 | 130.5 | 748.3 | 0.59 | 5.0 | 1.58 |

**Table S3.** Summary metrics of the sequence read processing. Two libraries were constructed. Raw reads were quality filtered (see methods). Any reads not including the primer sequences were discarded. Subsequently, reads were separated by sample using the attributed barcode giving unmerged paired-end reads. Reads were then merged discarding those pairs of low quality or not having an overlapping region. Locality codes for samples are presented in Methods: Sampling sites.

| ***Library 1*** | | | | | ***Library 2*** | | | | |
| --- | --- | --- | --- | --- | --- | --- | --- | --- | --- |
| ***Sample*** | **Raw Reads** | **Removed by QC** | **Unmerged** | **Merged** | **Sample** | **Raw Reads** | **Removed by QC** | **Unmerged** | **Merged** |
| *AKO-11* | 17827995 | 6850002 | 725524 | 706357 | AKO-21 | 21056050 | 8369999 | 543898 | 521645 |
| *AKO-12* |  |  | 624035 | 601468 | AKO-22 |  |  | 563531 | 530762 |
| *AKO-13* |  |  | 539653 | 499233 | AKO-23 |  |  | 648769 | 630689 |
| *CKY-11* |  |  | 590110 | 573431 | CKY-21 |  |  | 502092 | 472066 |
| *CKY-12* |  |  | 448392 | 426118 | CKY-22 |  |  | 614047 | 575873 |
| *CKY-13* |  |  | 243762 | 217567 | CKY-23 |  |  | 281736 | 265347 |
| *DHP-11* |  |  | 560540 | 547293 | DHP-21 |  |  | 634015 | 620121 |
| *DHP-12* |  |  | 455305 | 434091 | DHP-22 |  |  | 431940 | 414382 |
| *DHP-13* |  |  | 351919 | 337620 | DHP-23 |  |  | 513265 | 497179 |
| *GBI-11* |  |  | 452301 | 429627 | GBI-21 |  |  | 469135 | 444632 |
| *GBI-12* |  |  | 485084 | 454956 | GBI-22 |  |  | 623450 | 590069 |
| *GBI-13* |  |  | 508712 | 481863 | GBI-23 |  |  | 508364 | 482354 |
| *KDA-11* |  |  | 462640 | 440236 | KDA-21 |  |  | 545739 | 512934 |
| *KDA-12* |  |  | 408730 | 384139 | KDA-22 |  |  | 581009 | 552964 |
| *KDA-13* |  |  | 281619 | 259449 | KDA-23 |  |  | 361393 | 341134 |
| *KPI-11* |  |  | 476584 | 421159 | KPI-21 |  |  | 686422 | 618390 |
| *KPI-12* |  |  | 309553 | 294060 | KPI-22 |  |  | 574631 | 496557 |
| *KPI-13* |  |  | 400006 | 372175 | KPI-23 |  |  | 582376 | 566156 |
| *PBK-11* |  |  | 454885 | 414878 | PBK-21 |  |  | 512311 | 477140 |
| *PBK-12* |  |  | 460261 | 417068 | PBK-22 |  |  | 537018 | 510975 |
| *PBK-13* |  |  | 398143 | 379840 | PBK-23 |  |  | 506249 | 489322 |
| *TH-11* |  |  | 413287 | 388965 | TH-21 |  |  | 593446 | 554942 |
| *TH-12* |  |  | 409544 | 378506 | TH-22 |  |  | 404180 | 384248 |
| *TH-13* |  |  | 517404 | 472373 | TH-23 |  |  | 467035 | 450728 |

**Table S4:** Spearman correlation coefficients for the abundance of OTU count data and environmental variables. OTUs were differentially abundant between localities (FDR corrected Kruskal-Wallis test p < 0.05). The correlation estimators in bold were significant at p ≤ 0.001. All the OTUs in the table were assigned to the *Glomeraceae*.

|  | **OTU110** | **OTU13** | **OTU148** | **OTU28** | **OTU84** | **OTU85** |
| --- | --- | --- | --- | --- | --- | --- |
| **pH** | **-0.592** | **-0.489** | **-0.732** | **0.451** | **0.504** | **-0.659** |
| **OM** | -0.429 | -0.180 | **-0.489** | 0.272 | 0.153 | -0.445 |
| **P** | 0.018 | 0.004 | 0.144 | -0.171 | 0.124 | 0.213 |
| **K** | -0.198 | -0.236 | -0.402 | -0.030 | 0.112 | -0.328 |
| **Mg** | -0.450 | -0.310 | **-0.601** | **0.510** | 0.296 | **-0.482** |
| **Ca** | -0.160 | -0.375 | -0.305 | 0.250 | 0.179 | -0.129 |
| **Norg** | 0.144 | -0.146 | -0.171 | 0.010 | 0.045 | -0.067 |
| **C/N** | -0.341 | 0.026 | -0.088 | 0.145 | 0.052 | -0.119 |
| **Corg** | -0.436 | -0.180 | **-0.505** | 0.291 | 0.153 | **-0.461** |
| **bio01** | **0.464** | **0.536** | **0.638** | -0.370 | -0.418 | 0.402 |
| **bio02** | -0.443 | -0.330 | **-0.536** | **0.569** | 0.203 | **-0.455** |
| **bio03** | 0.265 | -0.399 | 0.067 | **-0.469** | 0.300 | 0.313 |
| **bio04** | -0.314 | 0.366 | -0.208 | **0.650** | -0.297 | -0.348 |
| **bio05** | **-0.461** | -0.285 | **-0.542** | **0.578** | 0.185 | **-0.477** |
| **bio06** | **0.467** | 0.342 | **0.511** | **-0.563** | -0.226 | 0.427 |
| **bio07** | **-0.463** | -0.284 | **-0.548** | **0.583** | 0.159 | **-0.475** |
| **bio08** | **0.464** | 0.448 | **0.572** | -0.438 | -0.311 | 0.403 |
| **bio09** | **0.473** | 0.395 | **0.561** | **-0.547** | -0.259 | 0.429 |
| **bio10** | 0.169 | **0.650** | 0.433 | 0.053 | **-0.534** | 0.186 |
| **bio11** | 0.442 | 0.372 | **0.626** | **-0.491** | -0.272 | **0.466** |
| **bio12** | **0.545** | 0.246 | **0.520** | -0.366 | -0.349 | **0.502** |
| **bio13** | 0.485 | 0.420 | 0.483 | -0.383 | -0.305 | 0.383 |
| **bio14** | 0.145 | -0.018 | 0.184 | -0.210 | 0.064 | 0.122 |
| **bio15** | 0.255 | **0.490** | 0.250 | -0.071 | -0.379 | 0.243 |
| **bio16** | 0.416 | **0.514** | **0.518** | -0.327 | -0.353 | 0.367 |
| **bio17** | **0.542** | 0.258 | **0.504** | **-0.586** | -0.193 | **0.526** |
| **bio18** | **0.476** | 0.038 | 0.144 | -0.106 | -0.243 | 0.387 |
| **bio19** | 0.129 | -0.306 | 0.050 | -0.240 | 0.160 | 0.056 |

**Table S5:** Spearman correlation coefficients for the abundance of AMF family count data and environmental variables. All families, other than the *Glomeraceae,* were differentially abundant among localities (FDR corrected Kruskal-Wallis test p < 0.05). The correlation estimators in bold were significant at < 0.001.

|  | ***Acaulosporaceae*** | ***Ambisporaceae*** | ***Archaeosporaceae*** | ***Geosiphonaceae*** | ***Pacisporaceae*** | ***Glomeraceae*** |
| --- | --- | --- | --- | --- | --- | --- |
| **pH** | 0.372 | -0.449 | **-0.538** | -0.154 | -0.154 | -0.265 |
| **OM** | 0.2 | -0.330 | -0.348 | -0.064 | -0.064 | -0.170 |
| **P** | 0.13 | -0.059 | 0.068 | -0.076 | -0.076 | -0.220 |
| **K** | 0.272 | -0.095 | -0.066 | 0.018 | 0.018 | -0.146 |
| **Mg** | 0.291 | **-0.452** | -0.439 | -0.332 | -0.332 | -0.204 |
| **Ca** | 0.282 | -0.266 | -0.320 | 0.054 | 0.054 | -0.225 |
| **Norg** | 0.067 | 0.005 | 0.049 | 0.294 | 0.294 | -0.006 |
| **C/N** | -0.004 | -0.182 | -0.207 | -0.281 | -0.281 | -0.055 |
| **Corg** | 0.201 | -0.330 | -0.352 | -0.075 | -0.075 | -0.164 |
| **bio01** | **-0.487** | 0.410 | **0.517** | 0.129 | 0.129 | 0.366 |
| **bio02** | 0.313 | **-0.538** | **-0.660** | -0.321 | -0.321 | -0.054 |
| **bio03** | 0.243 | 0.181 | 0.197 | 0.181 | 0.181 | -0.399 |
| **bio04** | -0.309 | -0.368 | -0.370 | -0.44 | -0.440 | **0.482** |
| **bio05** | 0.243 | **-0.550** | **-0.654** | -0.374 | -0.374 | -0.018 |
| **bio06** | -0.345 | **0.512** | **0.631** | 0.295 | 0.295 | 0.104 |
| **bio07** | 0.234 | **-0.538** | **-0.644** | -0.345 | -0.345 | 0.013 |
| **bio08** | -0.416 | **0.518** | **0.627** | 0.204 | 0.204 | 0.225 |
| **bio09** | -0.371 | **0.549** | **0.657** | 0.263 | 0.263 | 0.141 |
| **bio10** | **-0.607** | 0.075 | 0.199 | -0.181 | -0.181 | **0.556** |
| **bio11** | -0.327 | **0.475** | **0.600** | 0.289 | 0.289 | 0.124 |
| **bio12** | -0.157 | 0.309 | 0.343 | 0.322 | 0.322 | 0.180 |
| **bio13** | -0.299 | **0.462** | **0.560** | 0.240 | 0.240 | 0.224 |
| **bio14** | -0.042 | 0.159 | 0.118 | 0.174 | 0.174 | 0.023 |
| **bio15** | **-0.482** | 0.257 | 0.378 | -0.058 | -0.058 | 0.322 |
| **bio16** | -0.43 | **0.450** | **0.565** | 0.146 | 0.146 | 0.324 |
| **bio17** | -0.313 | **0.526** | **0.638** | 0.263 | 0.263 | 0.087 |
| **bio18** | -0.095 | 0.168 | 0.161 | -0.064 | -0.064 | 0.177 |
| **bio19** | 0.381 | 0.080 | -0.073 | 0.357 | 0.357 | -0.252 |
